# Computational Modeling of Hierarchically Polarized Groups by Structured Matrix Factorization

**DOI:** 10.3389/fdata.2021.729881

**Published:** 2021-12-22

**Authors:** Dachun Sun, Chaoqi Yang, Jinyang Li, Ruijie Wang, Shuochao Yao, Huajie Shao, Dongxin Liu, Shengzhong Liu, Tianshi Wang, Tarek F. Abdelzaher

**Affiliations:** ^1^ Computer Science Department, University of Illinois at Urbana-Champaign, Urbana, IL, United States; ^2^ Computer Science Department, George Mason University, Fairfax, VA, United States; ^3^ Computer Science Department, William and Mary, Williamsburg, VA, United States

**Keywords:** polarization, belief estimation, hierarchical, matrix factorization, unsupervised

## Abstract

The paper extends earlier work on modeling hierarchically polarized groups on social media. An algorithm is described that 1) detects points of *agreement* and *disagreement* between groups, and 2) divides them *hierarchically* to represent nested patterns of agreement and disagreement given a structural guide. For example, two opposing parties might disagree on core issues. Moreover, within a party, despite agreement on fundamentals, disagreement might occur on further details. We call such scenarios *hierarchically polarized groups*. An (enhanced) unsupervised Non-negative Matrix Factorization (NMF) algorithm is described for computational modeling of hierarchically polarized groups. It is enhanced with a language model, and with a proof of orthogonality of factorized components. We evaluate it on both synthetic and real-world datasets, demonstrating ability to hierarchically decompose overlapping beliefs. In the case where polarization is flat, we compare it to prior art and show that it outperforms state of the art approaches for polarization detection and stance separation. An ablation study further illustrates the value of individual components, including new enhancements.

## 1 Introduction

Extending a previous conference publication (Yang et al., 2020), this paper solves the problem of *unsupervised computational modeling of hierarchically polarized groups*. The model can accept, as input, a structural guide to the layout of groups and subgroups. The goal is to uncover the beliefs of each group and subgroup in that layout, divided into points of agreement and disagreement among the underlying communities and sub-communities, given their social media posts on polarizing topics. Most prior work clusters sources or beliefs into flat classes or stances (Küçük and Can, 2020). Instead, we focus on scenarios where the underlying social groups disagree on *some* issues but agree on others (i.e., their beliefs *overlap*). Moreover, we consider a (shallow) hierarchical structure, where communities can be further subdivided into subsets with their own agreement and disagreement points.

Our work is motivated, in part, by the increasing polarization on social media (Liu, 2012). Individuals tend to connect with like-minded sources (Bessi et al., 2016), ultimately producing echo-chambers (Bessi et al., 2016) and filter bubbles (Bakshy et al., 2015). Tools that could automatically extract social beliefs, and distinguish points of agreement and disagreement among them, might help generate future technologies (e.g., less biased search engines) that summarize information for consumption in a manner that gives individuals more control over (and better visibility into) the degree of bias in the information they consume.

The basic solution described in this paper is *unsupervised*. However, it does accept guidance on group/subgroup structure. Furthermore, the solution has an option for enhancement using prior knowledge of language models. By *unsupervised*, therefore, we mean that the (basic) approach does not need prior training, labeling, or remote supervision. This is in contrast, for example, to deep-learning solutions (Irsoy and Cardie, 2014; Liu et al., 2015; Wang et al., 2017) that usually require labeled data. The structural guidance, in this paper, is not meant to be obtained through training. Rather, it is meant as a mechanism for an analyst familiar with the situation to enter a template to match the inferred groups against. For example, the analyst might have the intuition that the community is divided into two conflicted factions, of which one is further divided into two subgroups with partial disagreement. They might be interested in understanding the current views of each faction/subgroup. The ability to exploit such analyst guidance (on the hierarchy of disagreement) is one of the distinguishing properties of our approach. In the absence of analyst intuitions, it is of course possible to skip structural guidance, as we show later in the paper. The basic algorithm can be configured so that it does not need language-specific prior knowledge (Liu, 2012; Hu Y. et al., 2013), distant-supervision (Srivatsa et al., 2012; Weninger et al., 2012), or prior embedding (Irsoy and Cardie, 2014; Liu et al., 2015), essentially making it *language-agnostic*. Instead, it relies on tokenization (the ability to separate individual words). Where applicable, however, we can utilize a *BERTweet* (Nguyen et al., 2020) variant that uses a pre-trained Tweet embedding to generate text features, if higher performance is desired. *BERTweet* is a specific language model for Tweets with the same structure as in the Bidirectional Encoder Representations from Transformers (BERT) (Devlin et al., 2019). While we test the solution only with English text, we conjecture that the its application can be easily extended to other languages (with the exception of those that do not have spaces between words, such as Chinese and Japanese, because we expect spaces as token separators). An advantage of unsupervised techniques is that they do not need to be retrained for new domains, jargon, or hash-tags.

The work is a significant generalization of approaches for polarization detection (Conover et al., 2011; Demartini et al., 2011; Bessi et al., 2016; Al Amin et al., 2017) that identify opposing positions in a debate but do not explicitly search for points of agreement. The unsupervised problem addressed in this paper is also different from unsupervised techniques for topic modeling (Litou and Kalogeraki, 2017; Ibrahim et al., 2018) and polarity detection (Al Amin et al., 2017; Cheng et al., 2017). Prior solutions to these problems aim to find *orthogonal* topic components (Cheng et al., 2017) or *conflicting* stances (Conover et al., 2011). In contrast, we aim to find components that adhere to a given (generic) *overlap* structure, presented as structural guidance from the user (e.g., from an analyst). Moreover, unlike solutions for hierarchical topic decomposition (Weninger et al., 2012; Zhang et al., 2018), we consider not only message content but also user attitudes towards it (e.g., who forwards it), thus allowing for better separation, because posts that share a specific stance are more likely to overlap in the target community (who end up spreading them).

This paper extends work originally presented at ASONAM 2020 (Yang et al., 2020). The extension augments the original paper in several aspects. First, we provide options for integrating a language model to improve outcomes. In this version, besides tokenization, we use language models that boost performance, compared to a purely lexical overlap-based approach. Second, we derive a new orthogonality property for the factorized components by our model. Finally, we conduct a simulation and new experiments that additionally verify our model in multiple scenarios involving both a flat group structure, and a hierarchical structure with complex sub-structure.

The work is evaluated using both synthetic data as well as real-life datasets, where it is compared to approaches that detect polarity by only considering who posted which claim (Al Amin et al., 2017), approaches that separate messages by content or sentiment analysis (Go et al., 2009), approaches that identify different communities in social hypergraphs (Zhou et al., 2007), and approaches that detect user stance by density-based feature clustering (Darwish et al., 2020). The results of this comparison show that our algorithm outperforms the state of the art. An ablation study further illustrates the impact of different design decisions on accomplishing this improvement.

The rest of the paper is organized as follows. Section 2 formulates the problem and summarizes the solution approach. Section 3 proposes our new belief structured matrix factorization model, and analyzes some model properties. Section 4 proves the property of orthogonality regarding one of the decomposed components. Section 5 and Section 6 present an experimental evaluation based on simulation and real data, respectively. We review the related literature on belief mining and matrix factorization in Section 7. The paper concludes with key observations and a statement on future directions in Section 8.

## 2 Problem Formulation

Consider an observed data set of posts collected from a social medium, such as Twitter, where each post is associated with a *source* and with semantic content, called a *claim*. Let S be the set of sources in our data set, and C be the set of claims made by those sources. While, in this paper, a claim is the content of a tweet (or retweet), the analytical treatment does not depend on this interpretation. Let matrix **X**, of dimension |S|×|C| be a matrix of binary entries denoting who claimed what. A claim can be made by multiple sources. In general, an entry of **X** could be a positive real number indicating a level of endorsement of a source to a claim. In this paper, we simplify by using binary entries; if source *S*_*i*_ endorsed claim *C*_*j*_, then *x*_*ij*_ = 1, otherwise *x*_*ij*_ = 0.

### 2.1 Problem Statement

We assume that the set of sources, S , is divided into a small number, *K*, of latent social groups, denoted by the subsets $G_{1}$ $G_{2}$ , …, $G_{K}$ , that form a tree. In this tree, group $G_{i}$ is a child (i.e., a subgroup) of $G_{k}$ if $G_{i}\subset G_{k}$ . Children of the same parent are disjoint groups. Members of group $G_{k}$ that do not belong to any of its children are denoted by the residual set $G_{k}^{-}$ . Within each group, $G_{k}$ , 1 ≤ *k* ≤ *K*, individuals have *shared beliefs* expressed by a set of claims. A shared belief of a group is a belief espoused by *all members* of the group. By definition, therefore, a child group inherits the shared beliefs of its parent. The child group may have additional shared beliefs within its group (not shared by the remaining members of the parent). Thus, we define the *incremental belief set*, $B_{k}$ , of group $G_{k}$ to be the beliefs held by group $G_{k}$ , beyond what’s inherited from its parent. The overall belief set of group $G_{k}$ is thus the union of incremental beliefs of its ancestors and itself. The problem addressed in this paper is to simultaneously 1) populate the membership of all latent groups, $G_{k}$ , in the tree, and 2) populate their incremental belief sets, $B_{k}$ , given a structural template, **B** (to be populated), that lays out the latent groups and latent belief sets.

**FIGURE 1:**
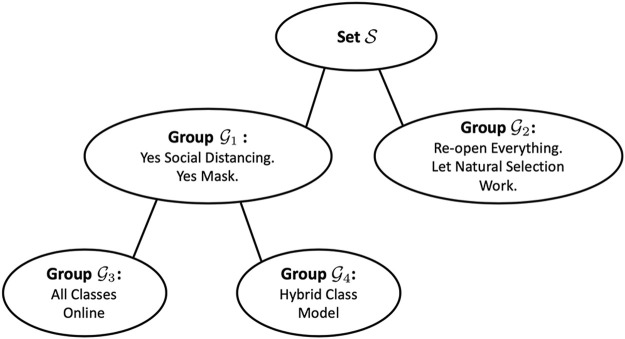
A notional example of hierarchical overlapping beliefs.

Figure 1 illustrates an example, inspired by the first wave of the COVID-19 pandemic in 2020. In this figure, a hypothetical community is divided on whether to maintain social distancing $\left(G_{1}\right)$ or reopen everything and let natural selection take place $\left(G_{2}\right)$ . Furthermore, while $G_{1}$ agree on social distancing, they disagree on some implementation details, such as whether classes should be entirely online $\left(G_{3}\right)$ or hybrid $\left(G_{4}\right)$ . In this example, an analyst might want to understand what groups/subgroups exist and what they disagree on. The analyst will enter a tree topology template, letting the algorithm populate who and which beliefs lie at which node of the tree.

### 2.2 Solution Approach

We use a non-negative matrix factorization algorithm to decompose the “who endorsed what” matrix, $X\in {\left\{0,1\right\}}^{\left|S|\times |C\right|}$ , into 1) a matrix, $U\in R_{+}^{|S|\times K}$ , that maps sources to latent groups, 2) a matrix, **B** ∈ {0,1}^*K*×*K*^, that maps latent groups to latent incremental belief sets (called the *belief structure matrix*), entered as structural guidance from the analyst, and 3) a matrix, $M\in R_{+}^{|C|\times K}$ , that maps claims to latent incremental belief sets. Importantly, since groups and belief sets are *latent*, the belief structure matrix, **B**, in essence, specifies the *latent structure* of the solution space that the algorithm needs to populate with specific sources and claims, thereby guiding factorization.

## 3 Structured Matrix Factorization

The proposed structured matrix factorization algorithms allows the user (e.g., an analyst) to specify matrix, **B**, to represent the relation between the latent groups, $G_{k}$ (that we wish to discover), and their incremental belief sets, $B_{k}$ (that we wish to discover as well). An element, *b*_*ij*_ of the matrix, **B**, is 1 if group $G_{i}$ adopts the belief set $B_{j}$ . Otherwise, it is zero. In a typical (non-overlapping) clustering or matrix factorization framework, there is an one-to-one correspondence between groups and belief sets, reducing **B** to an identity matrix. Structured matrix factorization extends that structure to an arbitrary relation. Matrix **B** can be thought of as a template relating latent groups (to be discovered) and belief sets (to be identified). It is a way to describe the structure that one wants the factorization algorithm to populate with appropriate group members and claims. While it might seem confusing to presuppose that one knows the latent structure, **B**, before the groups and belief sets in question are populated, below we show why this problem formulation is very useful.

### 3.1 An Illustrative Example

**FIGURE 2:**
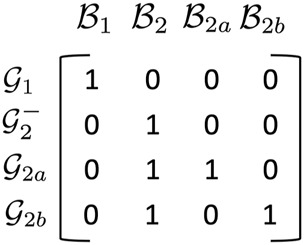
The belief structure matrix, **B**.

Consider a conflict involving two opposing groups, say, a minority group $G_{1}$ and a majority $G_{2}$ . Their incremental belief sets are denoted by $B_{1}$ and $B_{2}$ , respectively. The two groups disagree on everything. Thus, sets $B_{1}$ and $B_{2}$ do not overlap. An unfriendly agent wants to weaken the majority and conjectures that the majority group might disagree on something internally. Thus, the unfriendly postulates that group $G_{2}$ is predominantly made of subgroups $G_{2a}$ and $G_{2b}$ . While both subgroups agree on the shared beliefs, $B_{2}$ , each subgroup has its own incremental belief sets, $B_{2a}$ and $B_{2b}$ , respectively. The structure matrix in Figure 2 represents the belief structure postulated above.

For example, the second column indicates that the belief set $B_{2}$ is shared by all members of group $G_{2}$ (hence, there is a “1” in rows of subgroups $G_{2a}$ , $G_{2b}$ , and the residual $G_{2}^{-}$ ). The third and fourth columns state that belief sets $B_{2a}$ and $B_{2b}$ are unique to subgroups $G_{2a}$ and $G_{2b}$ , respectively. Note how the beliefs espoused by different groups overlap. For example, from the third and fourth rows, we see that groups $G_{2a}$ and $G_{2b}$ overlap in the set of beliefs, $B_{2}$ .

An interesting question might be: which sources belong to which group/subgroup? What are the incremental belief sets $B_{2a}$ and $B_{2b}$ that divide group $G_{2}$ (i.e., are shared only by the individual respective subgroups)? What are the shared beliefs $B_{2}$ that unite it? What are the beliefs, $B_{1}$ , of group $G_{1}$ ? These are the questions answered by our structured matrix factorization algorithm whose input is (only) matrix, **X**, and matrix, **B** (Figure 2).

### 3.2 Mathematical Formulation

To formulate the hierarchical overlapping belief estimation problem, we introduce the notion of claim *endorsement*. A source is said to *endorse* a claim if the source finds the claim agreeable with their belief. Endorsement, in this paper, represents a state of *belief*, not a physical act. A source might find a claim agreeable with their belief, even if the source did not explicitly post it. Let the probability that source *S*_*i*_ endorses claim *C*_*j*_ be denoted by   Pr(*S*_*i*_ *C*_*j*_). We further denote the proposition $S_{i}\in G_{p}$ by $S_{i}^{p}$ , and the proposition $C_{j}\in B_{q}$ by $C_{j}^{q}$ . Thus, $\mathrm{Pr}\left(S_{i}^{p}\right)$ denotes the probability that source $S_{i}\in G_{p}$ . Similarly, $\mathrm{Pr}\left(C_{j}^{q}\right)$ denotes the probability that claim $C_{j}\in B_{q}$ . Following the law of total probability:

$$\mathrm{Pr}\left(S_{i}C_{j}\right)=\sum_{p,q}\mathrm{Pr}\left(S_{i}C_{j}|S_{i}^{p}C_{j}^{q}\right)\mathrm{Pr}\left(S_{i}^{p}C_{j}^{q}\right)=\sum_{p,q}\mathrm{Pr}\left(S_{i}C_{j}|S_{i}^{p}C_{j}^{q}\right)\mathrm{Pr}\left(S_{i}^{p}\right)\mathrm{Pr}\left(C_{j}^{q}\right).$$ (1)

By definition of the belief structure matrix, **B**, we say that $\mathrm{Pr}\left(S_{i}C_{j}|S_{i}^{p}C_{j}^{q}\right)=1$ if *b*_*pq*_ = 1 in the belief structure matrix. Otherwise, $\mathrm{Pr}\left(S_{i}C_{j}|S_{i}^{p}C_{j}^{q}\right)=0$ . Let $u_{ip}=\mathrm{Pr}\left(S_{i}^{p}\right)$ and $m_{jq}=\mathrm{Pr}\left(C_{j}^{q}\right)$ . Let *u*_*i*_ and *m*_*j*_ be the corresponding vectors, with elements ranging over values of *p* and *q* respectively. Thus, we get: $\mathrm{Pr}\left(S_{i}C_{j}\right)=u_{i}^{⊤}Bm_{j}$ . Let the matrix **X** ^*G*^ be the matrix of probabilities,  Pr(*S*_*i*_ *C*_*j*_), such that element $x_{ij}^{G}=\mathrm{Pr}\left(S_{i}C_{j}\right)$ . Thus:

$$X^{G}=UBM^{⊤}$$ (2)

where **U** is a matrix whose elements are *u*_*ip*_ and **M** is a matrix whose elements are *m*_*jq*_. Factorizing **X** ^*G*^, given **B**, would directly yield **U** and **M**, whose elements are the probabilities we want: elements of matrix **U** yield the probabilities that a given source *S*_*i*_ belongs to a given group $G_{p}$ , whereas elements of matrix **M** yield the probabilities that a claim *C*_*j*_ belongs to a belief $B_{q}$ . Each source is then assigned to a group and each claim to a belief set, based on the highest probability entry in their row of matrix **U** and **M**, respectively. In practice, **B** could be customized up to certain tree depth to meet different granularity of belief estimation.

### 3.3 Estimating **X** ^*G*^

Unfortunately, we do not really have matrix **X** ^*G*^ to do the above factorization. Instead, we have the *observed* source-claim matrix **X** that is merely a sampling of what the sources endorse. (It is a sampling because a silent source might be in agreement with a claim even if they did not say so.) Using **X** directly is suboptimal because it is very sparse. It is desired to estimate that a source endorses a claim even if the source remains silent. We do so on two steps.

#### 3.3.1 Message Interpolation (the M-Module)

First, while source *S*_*i*_ might have not posted a specific claim, *C*_*j*_, it may have posted similar ones. If a source $S_{i}$ posted, retweeted, or liked claim $C_{j}$ in our data set (i.e., *x*_*ij*_ = 1 in matrix **X**), then we know that the source endorses that claim (i.e., $x_{ij}^{M}=1$ in matrix **X** ^*M*^). The question is, what to do when *x*_*ij*_ = 0? In other words, we need to estimate the likelihood that the source endorses a claim, when no explicit observations of such endorsement were made. We do so by considering the claim similarity matrix **D**, of dimension |C|×|C| . If source $S_{i}$ was observed to endorse claims $C_{k}$ similar to $C_{j}$ , then it will likely endorse $C_{j}$ with a probability that depends on the degree of similarity between $C_{j}$ and $C_{k}$ . Thus, when *x*_*ij*_ = 0, we can estimate $x_{ij}^{M}$ by weighted sum interpolation:

$$x_{ij}^{M}=\underset{k:\;x_{ik}\neq 0}{\sum }d_{kj}\cdot x_{ik}$$ (3)

To compute matrix **D**, in this work, we provide two configurations. For the language agnostic approach, we first compute a bag-of-words (BOW) vector *w*_*j*_ for each claim *j*, and then normalize it using vector *L*_2_ norm, $\bar{w_{j}}=w_{j}/\|w_{j}{\|}_{2}$ . For the *BERTweet* approach, *w*_*j*_ is the embedding for each claim. We select non-zero entries *x*_*ij*_ in each row *i* of **X** as *medoids* $\left\{\bar{w_{j}}|x_{ij}\neq 0\right\}$ . We assume that claims close to any of the *medoids* could also be endorsed by $S_{i}$ as well. Based on that, we use $d_{kj}=\phi \left(\left\|\bar{w_{j}}-\bar{w_{k}}\right\|\right)$ in Eq. 3. A Gaussian radial basis function (RBF) is used for $\phi \left(r\right)=e^{-{\left(\epsilon r\right)}^{2}}$ . This is called *message interpolation* (the M-module). The output of this module is a matrix **X** ^*M*^. If the resulting value of $x_{ij}^{M}$ is less than 0.2, we regard that it is far from all of the *medoids* and set it back to 0. In the experiments presented in our evaluation, *ϵ* is set to 0.5 for the synthetic dataset and 0.05 for each of the *United States Election 2020*, *Eurovision 2016*, and *Global Warming* datasets.

#### 3.3.2 Social Graph Convolution (S-Module)

To further improve our estimation of matrix **X** ^*G*^, we assume that sources generally hold similar beliefs to those in their immediate social neighborhood. Thus, we perform a smoothing of matrix **X** ^*M*^ by replacing each cell, *x*_*ij*_, by a weighted average of itself and the entries pertaining to neighbors of its source, *S*_*i*_, in the social graph. Let matrix **A**, of dimension |S|×|S| , denote the social graph. Each entry, *a*_*ij*_, denotes the influence of user *S*_*i*_ on user *S*_*j*_. **A** is thus the adjacency matrix of a social network graph. In this paper, we construct **A** by calculating the frequency of each source *S*_*i*_ retweeting posts of source *S*_*j*_. We call it the *retweet graph*. The update of entries of **X** ^*M*^ by smoothing over entries of neighboring sources is called *social graph convolution* (S-module). It results in an improved estimate, **X** ^*MS*^.

More specifically, from the social dependency matrix **A** (user-user retweet frequency), we can compute the degree matrix **F** by summing each row of **A**. The random walk normalized adjacency is denoted as $A_{rw}^{\sim }=F^{-1}A$ . We define our propagation operator based on $A_{rw}^{\sim }$ with self-loop re-normalization, $A_{rw}^{¯}\leftarrow \frac{1}{2}{F_{rw}^{\sim }}^{-1}\left(A_{rw}^{\sim }+I\right)$ . Thus, the new source-claim network is given by,

$$X^{MS}=A_{rw}^{¯}X^{M},$$ (4)

where each row of $A_{rw}^{¯}$ adds up to 1. The effect of the propagation operator is to convolve the information from 1-hop neighbors, while preserving half of the information from itself. Note that, we deem dependency beyond 1-hop too weak to capture, so we do not consider **A** ^*n*^, where *n* > 1. From a macroscopic perspective, this social graph convolution recovers some of the possible source-claim connections and also enforces the smoothness of matrix **X** ^*MS*^. We can now take, **X** ^*G*^ ≈ **X** ^*MS*^, and decompose it as presented in Section 3.2, Eq. 2, with *L*_1_ and *L*_2_ regularizations to enforce sparsity and prevent overfitting.

### 3.4 Loss and Optimization

Given a belief mixture matrix, **B**, we now factorize **X** ^*MS*^ to estimate matrices **U** and **M** that decide the belief regions associated with sources and claims, respectively. (e.g., the estimated belief for claim $C_{j}$ is given by the index of maximum entry in the *j*_*th*_ row of **M**).

*Regularization.* To avoid model overfitting, we include widely used *L*_2_ regularization. Also, we enforce the sparsity of **U** and **M** by introducing an *L*_1_ norm. The overall objective function becomes (defined by the Frobenius-norm),

$$J=\|X^{MS}-{UBM}^{⊤}{\|}_{F}^{2}+\lambda_{1}\|U{\|}_{F}^{2}+\lambda_{1}\|M{\|}_{F}^{2}+\lambda_{2}\|U{\|}_{1}+\lambda_{2}\|M{\|}_{1}.$$ (5)

We rewrite *J* using matrix trace function *tr*(⋅)

$$\begin{array}{ll} J & =tr\left(X^{MS^{⊤}}X^{MS}\right)-2tr\left(X^{MS^{⊤}}{UBM}^{⊤}\right)+tr\left({UBM}^{⊤}{MB}^{⊤}U^{⊤}\right)\\ & +\lambda_{1}tr\left(U^{⊤}U\right)+\lambda_{1}tr\left(M^{⊤}M\right)+\lambda_{2}\|U{\|}_{1}+\lambda_{2}\|M{\|}_{1}. \end{array}$$ (6)

We minimize *J* by gradient descent. Since only the non-negative region is of interest, derivatives of the *L*_1_ norm are differentiable in this setting. By referring to the gradient of traces of product with constant matrix **A**, ∇_**X**_ *tr* (**AX**) = **A** ^*⊤*^ and ∇_**X**_ *tr* (**XAX** ^*⊤*^) = **X** (**A+ A** ^*⊤*^), the partial derivative of *J* w.r.t. **U** and **M** are calculated as,

$$\begin{array}{ll} & \nabla_{U}=-2X^{MS}{MB}^{⊤}+2{UBM}^{⊤}{MB}^{⊤}+2\lambda_{1}U+\lambda_{2}1,\\ & \nabla_{M}=-2X^{MS^{⊤}}UB+2{MB}^{⊤}U^{⊤}UB+2\lambda_{1}M+\lambda_{2}1. \end{array}$$

The gradient matrix ∇_**U**_ is of dimension |S|×K , and ∇_**M**_ is of dimension |C|×K . Estimation step begins by updating **U** ←**U** − *η*∇_**U**_ and **M** ←**M** − *η*∇_**M**_, and *η* is the step size. Negative values might appear in the learning process, which are physically meaningless in this problem. Thus, we impose non-negativity constraints for **U** and **M** during the update. A *ReLU*-like strategy is utilized: when any entry of **U** or **M** becomes negative, it is set to be *ξ*. In the experiment, we set *ξ* = 10^−8^, *λ*_1_ = *λ*_2_ = 10^−3^. Note that the initial entry values of **U** and **M** are randomized uniformly from (0, 1).

Algorithm 1 : Belief Structured Matrix Factorization (BSMF).

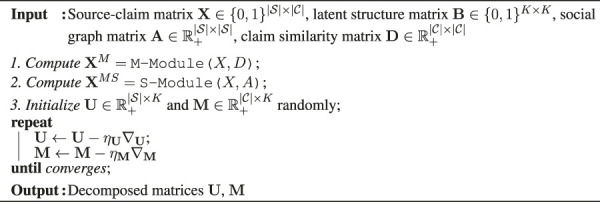

#### 3.4.1 Run-time Complexity

During the estimation, we generalize standard NMF multiplicative update rules (Lee and Seung, 2001) for our tri-factorization,

$$\eta_{U}=\frac{1}{2}\frac{U}{{UBM}^{⊤}{MB}^{⊤}},\;\;\eta_{M}=\frac{1}{2}\frac{M}{{MB}^{⊤}U^{⊤}UB}.$$ (7)

**FIGURE 3:**
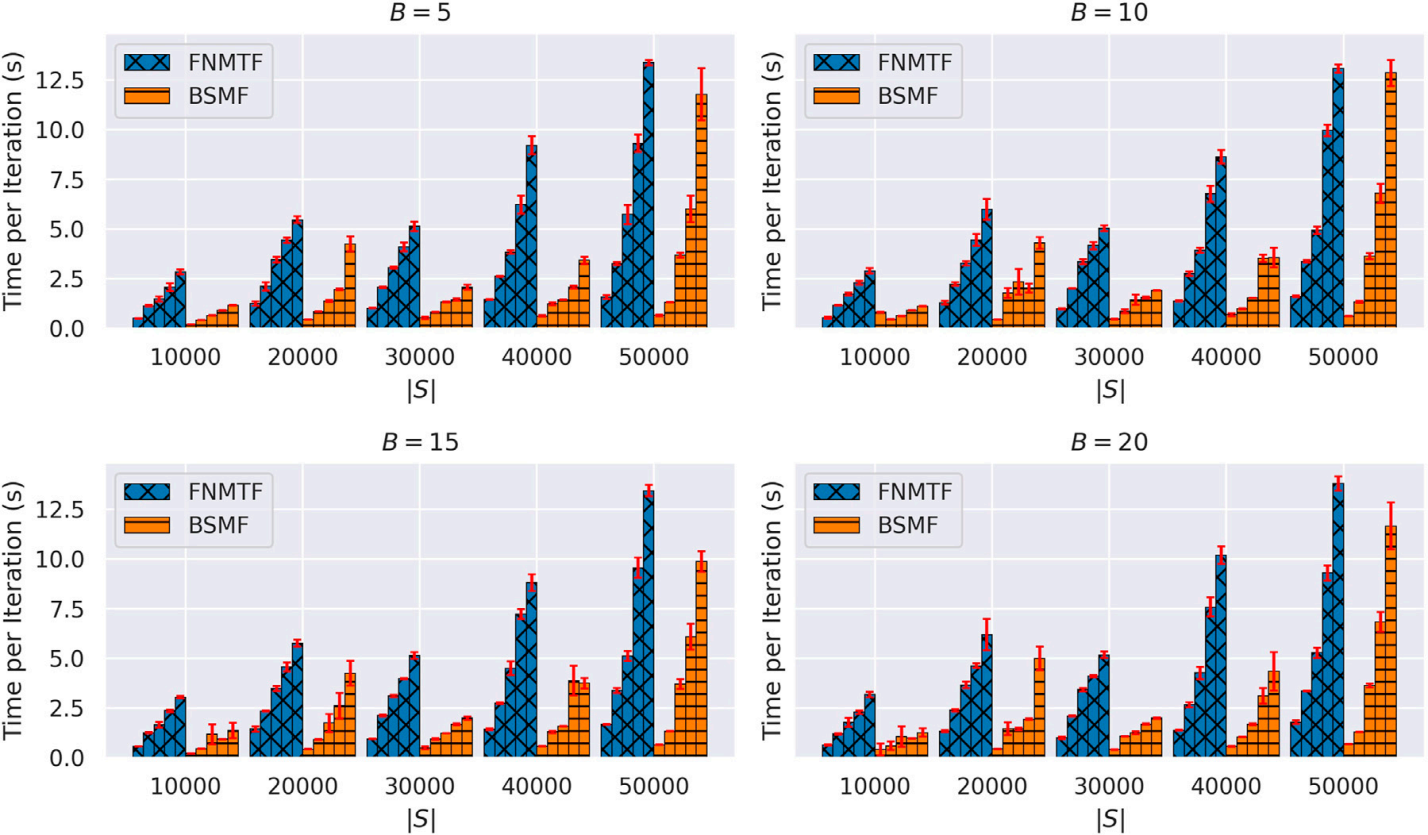
Running time under different configurations of dimensions of **X** and **B**. For each method, under the same |*S*|, |*C*| takes 10000, 20000, 30000, 40000, 50000 respectively. Red bar indicates standard deviation on time measurements.

Note that, *K* (number of belief groups) is picked according to the dataset, and it typically satisfies K≪min(|S|,|C|) . Algorithmically, updating **U** and **M** takes $O\left(K|S\|C|\right)$ per iteration, similar to the typical NMF. The number of iterations before the empirical convergence is usually no more than 200 for random initialization in our experiments, and thus we claim that our model is scalable and efficient. In Figure 3, we present our measurements on time taken per iteration for our algorithm and FNMTF (Wang et al., 2011), which is known to be able to perform tri-factorization fast. We measured each iteration 20 times to make the results more accurate. Our algorithm, with the generalized multiplicative update rules, performs better. This is because our algorithm does not need to update the **B** matrix, and avoids using matrix inversion. The graph appears to be consistent with our time complexity analysis.

## 4 Property of Orthogonality

The basic idea of belief structured matrix factorization (BSMF) is to decompose **X** ^*MS*^ ≈ **UBM** ^*⊤*^. What does that imply regarding the generated rows of decomposition matrices? Below, we show that the rows of matrix **U** are approximately orthogonal and that the rows of matrix **M** are also approximately orthogonal. This property suggests a sense of decomposition quality in that the latent factors produced constitute more efficient (non-redundant) encoding bases of the original information.

Theorem 1. *After BSMF factorization,* {*U*_*k*_} *are approximately orthogonal.*

PROOF. We start the analysis by the recapitulation of loss function,

$$\begin{array}{ll} J & =\|X^{MS}-{UBM}^{⊤}{\|}_{F}^{2}+\lambda_{1}\|U{\|}_{F}^{2}+\lambda_{1}\|M{\|}_{F}^{2}+\lambda_{2}\|U{\|}_{1}+\lambda_{2}\|M{\|}_{1}.\\ & =tr\left(X^{MS^{⊤}}X^{MS}\right)-2tr\left(X^{{MS}^{⊤}}{UBM}^{⊤}\right)+tr\left({UBM}^{⊤}{MB}^{⊤}U^{⊤}\right)\\ & \;+\lambda_{1}tr\left(U^{⊤}U\right)+\lambda_{1}tr\left(M^{⊤}M\right)+\lambda_{2}\|U{\|}_{1}+\lambda_{2}\|M{\|}_{1}. \end{array}$$ (8)

Let us first ignore the *L*_1_ and *L*_2_ term. Since $tr\left({X^{MS}}^{⊤}X^{MS}\right)$ is a constant, the problem of minimizing Eq. 8 is equivalent to,

$$\underset{U,M\geq 0}{\max}tr\left({X^{MS}}^{⊤}{UBM}^{⊤}\right),$$ (9)

$$\text{and}\;\;\;\underset{U,M\geq 0}{\min}tr\left({UBM}^{⊤}{MB}^{⊤}U^{⊤}\right).$$ (10)

The first objective, Eq. 9, is similar to K-means type clustering after expanding the trace function, which maximize within-cluster similarities,

$$\max\underset{i,j}{\sum }u_{i}^{⊤}Bm_{j}x_{ij}.$$ (11)

The second objective is to enforce orthogonality approximately. Because **UBM** ^*⊤*^ ≈ **X** ^*MS*^, let us add *L*_2_-norm of **U** and **M** now, so that the scale of **U**, **M** are constrained Wang and Zhang (2012). Since **B** is positive and fixed, $tr\left(U^{⊤}U\right)=\|U{\|}_{F}^{2}$ and $tr\left({BM}^{⊤}{MB}^{⊤}\right)=\|{MB}^{⊤}{\|}_{F}^{2}\leq \|M{\|}_{F}^{2}\|B{\|}_{F}^{2}$ are approximately constants. So Eq. 10 becomes,

$$\underset{U,M\geq 0}{\min}tr\left(\left(U^{⊤}U-I\right)\left({BM}^{⊤}{MB}^{⊤}-I\right)\right).$$ (12)

By Cauchy’s inequality, we further have,

$$\begin{array}{ll} & tr\left(\left(U^{⊤}U-I\right)\left({BM}^{⊤}{MB}^{⊤}-I\right)\right)\\ =\; & \underset{i=1,\ldots ,K;\;j=1,\ldots ,K}{\sum }{\left(U^{⊤}U-I\right)}_{ij}{\left({BM}^{⊤}{MB}^{⊤}-I\right)}_{ji}\\ \leq \; & \|U^{⊤}U-I{\|}_{F}\cdot \|{BM}^{⊤}{MB}^{⊤}-I{\|}_{F}. \end{array}$$ (13)

Thus, the overall second objective is bounded by,

$$\begin{array}{ll} & \underset{U,M\geq 0}{\min}\|U^{⊤}U-I{\|}_{F}\cdot \|{BM}^{⊤}{MB}^{⊤}-I{\|}_{F}\\ \Rightarrow \; & \underset{U\geq 0}{\min}\|U^{⊤}U-I{\|}_{F}\cdot \underset{M\geq 0}{\min}\|{BM}^{⊤}{MB}^{⊤}-I{\|}_{F}. \end{array}$$ (14)

which enforces the orthogonality of rows in set {*U*_*k*_}.

Corollary 1.1. *After BSMF factorization,* {*M*_*k*_} *are approximately orthogonal.*

PROOF Similar to the proof above, $tr\left(M^{⊤}M\right)=\|M{\|}_{F}^{2}$ and $tr\left(B^{⊤}U^{⊤}UB\right)=\|UB{\|}_{F}^{2}\leq \|U{\|}_{F}^{2}\|B{\|}_{F}^{2}$ are approximately constants. Equation 10 becomes,

$$\underset{U,M\geq 0}{\min}tr\left(\left(M^{⊤}M-I\right)\left(B^{⊤}U^{⊤}UB-I\right)\right).$$ (15)

Thus, the overall second objective is bounded by,

$$\underset{M\geq 0}{\min}\|M^{⊤}M-I{\|}_{F}\cdot \underset{U\geq 0}{\min}\|B^{⊤}U^{⊤}UB-I{\|}_{F}.$$ (16)

which enforces the orthogonality of pre-belief bases {*M*_*k*_}.

In other words, the generated groups into which all sources and claims decomposed are maximally diverse and are not redundant in some sense.

## 5 Illustrative Simulation

To help visualize the belief structure developed in our approach and offer a concrete example of approximate orthogonality of groups (i.e., pre-belief bases), we created simulations to evaluate our models multiple times against two simpler variants. We also provide 3D visualizations of the factorized components to observe their spatial characteristics.

### 5.1 Dataset Construction

To offer a simplified and controllable setting in which we can visualize the novel factorization algorithm, we build a synthetic dataset, where two groups of users are created, a minority, $G_{1}$ (100 users) of belief set $B_{1}$ , and a majority, $G_{2}$ (300 users) of belief set $B_{2}$ . The majority includes two subgroups, $G_{2a}$ and $G_{2b}$ (100 users each) of incremental belief sets $B_{2a}$ and $B_{2b}$ , respectively. Essentially, the groups follow the hierarchical structure illustrated in Figure 2. For each group, we built disjoint claim corpora, denoted by *c*_1_, *c*_2_, *c*_2*a*_ and *c*_2*b*_ to express their respective belief sets. Users were simulated to post claims chosen from their group’s assigned corpus or from their parent’s corpus (we randomly generate 20 claims for each user). Thus, for example, users in group $G_{2a}$ could post claims generated from *c*_2*a*_ or from the parent corpus *c*_2_, but users in group $G_{1}$ only post claims from corpus *c*_1_. In sum, 400 users and 8,000 claims were created. To keep it simple, in this experiment, we do not impose social relations. Instead, we use the identity matrix for the adjacency **A**.

### 5.2 Method Comparison

The factorization algorithm uses the belief structure matrix in Figure 2. Two simpler variants are introduced: 1) the first variant substitutes **B** with an identity matrix, and takes a standard NMF formulation **X** ^*G*^ = **UM** ^*⊤*^; 2) the second variant substitutes **B** with an learnable matrix $\tilde{B}$ , which takes a standard non-negative matrix tri-factorization (NMTF) form, $X^{G}=U\tilde{B}M^{⊤}$ . Obviously, NMTF offers more freedom. However, the need to learn parameters of matrix $\tilde{B}$ can cause overfitting. We use the same regularization settings for NMF, NMTF and our BSMF to make sure the comparison is fair. Empirically, after 150 ∼ 200 iterations, all three methods converge. The predicted belief set label for each claim is given by the index of the maximum value in this final representation from matrix, **M**.^1^

### 5.3 Results of 200 Rounds

We run each model for 200 times and compute the classification accuracy of users and claims for each algorithm in every run, then average them over all runs. We find that BSMF consistently outperforms NMF and NMTF. The average values of accuracy for BSMF, NMF and NMTF are 97.34*%*, 93.78*%*, 95.54*%*, respectively. As might be expected, specifying matrix, **B**, guides subsequent factorization to a better result compared to both NMF and NMTF.

### 5.4 Visualization of Results

**FIGURE 4:**
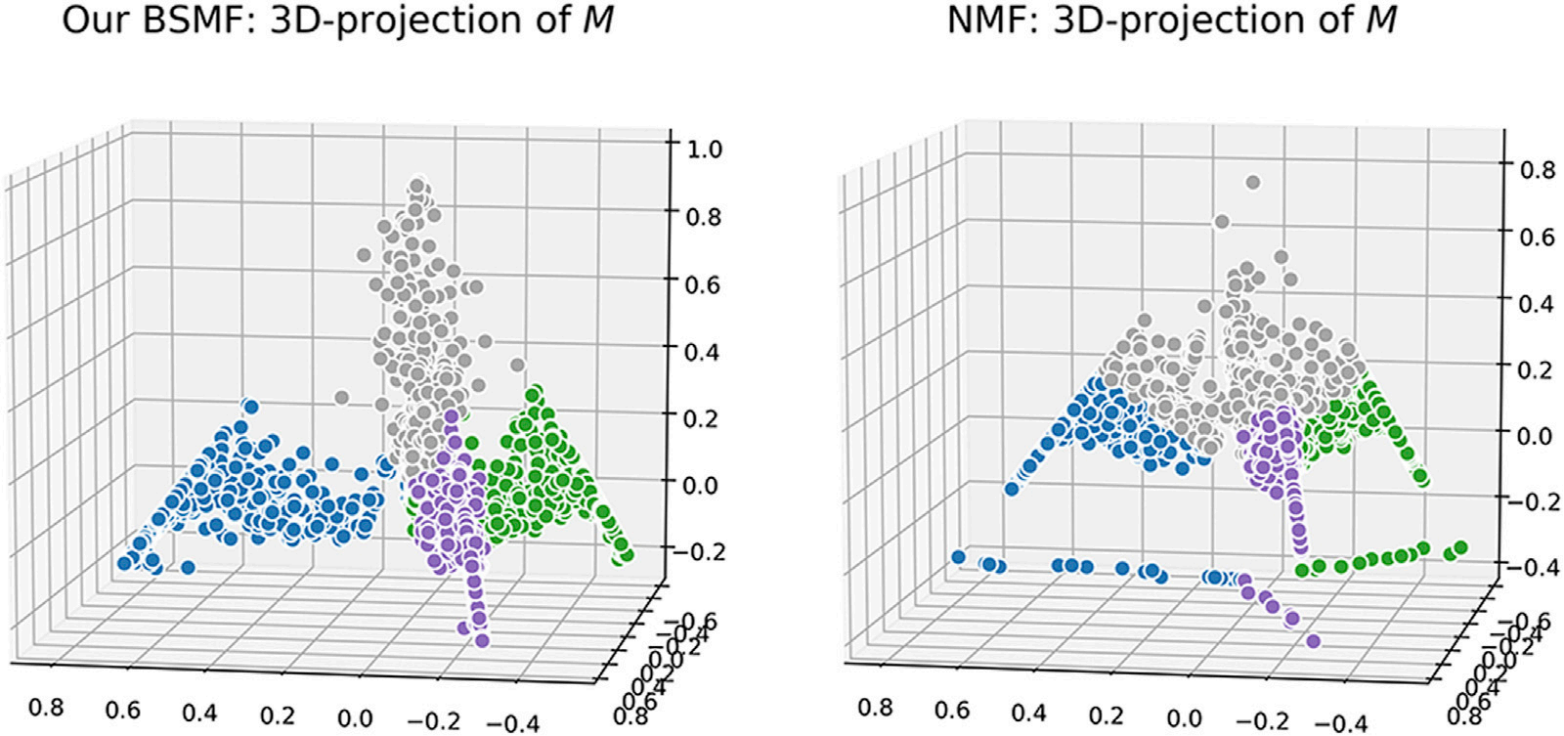
Visualization of *M* for our BSMF **(left)** and NMF **(right)**.

**FIGURE 5:**
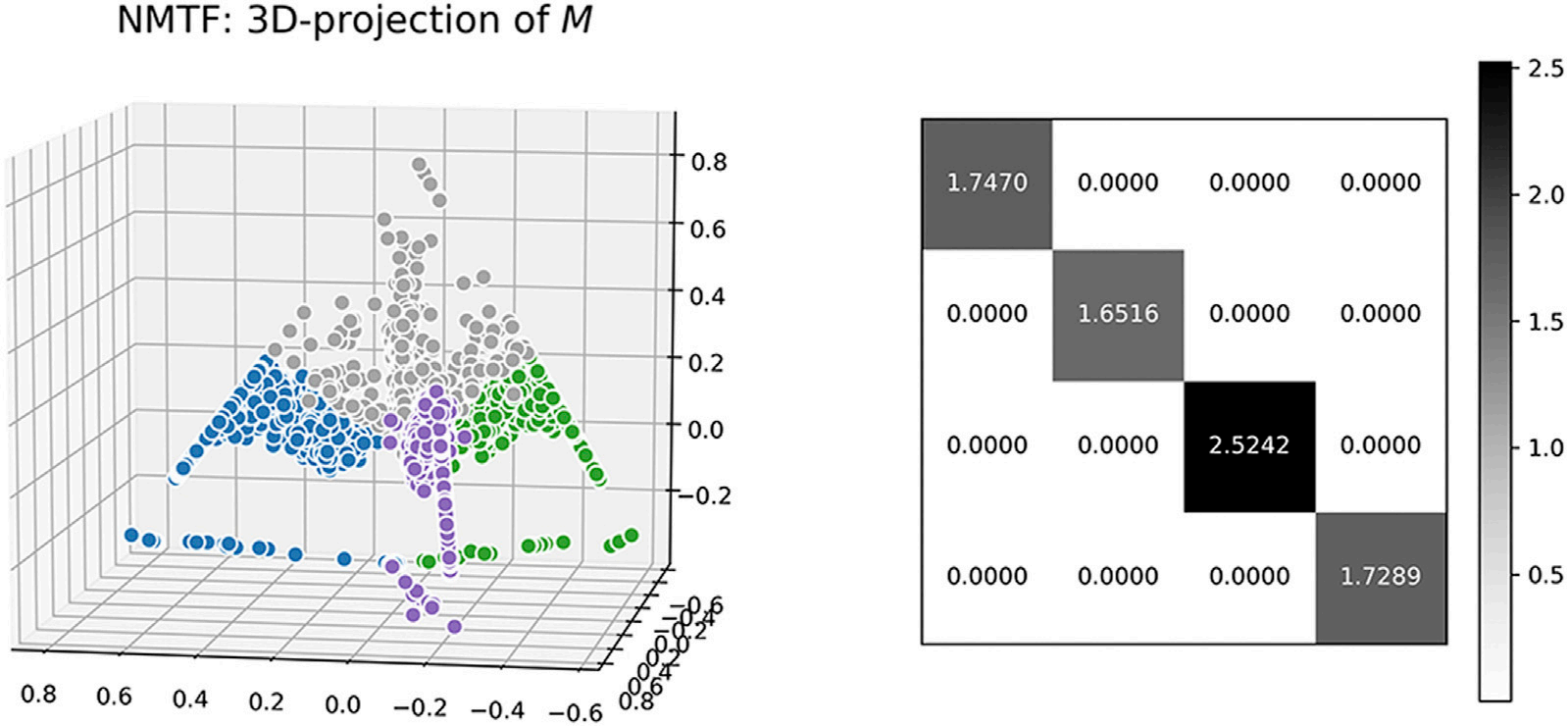
Visualization of *M* **(left**) and $\tilde{B}$ **(right)** for NMTF.

We also visualize the computed matrix, **M**, for each algorithm. Colors are based on ground-truth labels. In Figure 4 and Figure 5, we project the estimated **M** into a 3-D space, where each data point represents a message. In each figure, all of the data points seem to lie in a regular tetrahedron (should be regular *K*-polyhedron for more general *K*-belief cases). It is interesting that for NMF, different colors are very closely collocated in the latent space (e.g., there is very little separation between the grey color and others). It is obviously difficult to draw a boundary for the crowded mass. NMTF is a little bit better: different colors are visually more separable. We also visualize the learned $\tilde{B}\in R^{4\times 4}$ , which turns out to be an SVD-like diagonal matrix, meaning that pure NMTF only learns the independent variances aligned with each belief.

The projection result of BSMF are different: different color points are better separated and grouped by colors. We hypothesize that in the four-dimensional space, data points should be perfectly aligned with one of the belief bases/parts, and these four bases are conceivably orthogonal in that space. In a word, the results on synthetic data strongly suggest that our model disentangles the latent manifold leading to a better separation of messages by belief sets.

## 6 Experiments With Empirical Data

In the section, we evaluate *Belief Structured Matrix Factorization (BSMF)* using real-world Twitter datasets of different patterns of belief overlap, hierarchy, or disjointedness. Key statistics of these data sets are briefly summarized in Table 1. Our model is compared to (up to) six baselines and five model variants. Each experiment is run 20 times to acquire the mean and the standard deviation for measurements. We elaborate the experimental settings and results below.

**TABLE 1:** Basic statistics for three twitter datasets.

Dataset	# Sources	# Claims	# All tweets	# Retweets
Eurovision2016	3,514	5,812	9,868	6,001
US Election 2020	502	4,159	7,030	6,942
Global Warming	14,752	7,030	16,418	9,341

### 6.1 Disjoint Belief Structure: *United States Election 2020*

We start with a dataset where we posit that agreements are too weak to the point where beliefs of key groups are assumed to be “disjoint” (i.e., share no overlap). Specifically, during the 2020 United States election, messages on social media tended to be very polarized, either supporting the democratic or the republican party, exclusively. The example demonstrates a simplest case of belief factorization for clarity.

#### 6.1.1 Dataset

We use Apollo Social Sensing Toolkit^2^ to collect the United States Election 2020 dataset. The dataset contains tweets collected during the United States Election in 2020, where the support for candidates was split between former president Donald Trump and president Joe Biden. Basic statistics are reported in Table 1. Overall, the most retweeted 237 tweets from the dataset are manually annotated, which separate into 133 pro-Trump and 104 anti-Trump claims.

In this dataset, the sources are split into two groups $G_{1}$ and $G_{2}$ , each with belief sets, $B_{1}$ and $B_{2}$ , respectively. The belief structure matrix, therefore, is:

$$B=\left[\begin{array}{cc} 1 & 0\\ 0 & 1 \end{array}\right]$$

where rows correspond to groups $G_{1}$ and $G_{2}$ , respectively, and columns correspond to belief sets, $B_{1}$ and $B_{2}$ , respectively.

#### 6.1.2 Baselines

We select six baseline methods that encompass different perspectives on belief separation:

• Ra*ndom*: A trivial baseline that annotates posts randomly, giving equal probability to each label.
• DB*SCAN* (Darwish et al., 2020): A density-based clustering technique that extracts user-level features and performs DBSCAN for unsupervised user stance detection in Twitter. In this paper, we use DBSCAN and then map the user stance to claim stances with majority voting, as a baseline.
• Sen*timent140* (Go et al., 2009): Content-aware solutions based on language or sentiment models. In the implementation, each of the claims is a query through Sentiment140 API, which responds with a polarity score. The API will respond with the same score upon multiple same requests.
• H-N*Cut* (Zhou et al., 2007): The method views the bipartite structure of the source-claim network as a hypergraph, where claims are nodes and sources are hyperedges. The problem is thus seen as a hypergraph community detection problem, where community nodes represent posts. We implement *H-NCut*, a hypergraph normalized cut algorithm.
• Po*larization* (Al Amin et al., 2017): A baseline that uses an NMF-based solution for social network belief extraction to separate biased and neutral claims.
• NM*TF*: A baseline with a learnable mixture matrix. We compare our model with it to demonstrate that pure learning without a prior is not enough to unveil the true belief overlap structure in real-world applications.
• FNM*TF* (Wang et al., 2011): A baseline for data co-clustering with non-negative matrix tri-factorization. We compare to this model mainly to have a run-time complexity comparison and demonstrate the importance of structural guidance.

Different variants of BSMF are also evaluated to verify the effectiveness of message similarity interpolation (the M-module) and social graph convolution (the S-module). BSMF_*MS*_ incorporates both modules. Models with only the M-module or the S-module are named BSMF_*M*_ and BSMF_*S*_, respectively. BSMF denotes the model without either module. BSMF_*MS*−*BERT*_ and BSMF_*M*−*BERT*_ are two variants whose M-modules are configured to use *BERTweet* (Nguyen et al., 2020) embeddings, while BSMF_*MS*_ and BSMF_*M*_ are using lexical overlap enabled by tokenization.

#### 6.1.3 Evaluation Metrics

We evaluate claim separation, since only claim labels are accessible. We use the *Python scikit-learn* package to help with the evaluation. Multiple metrics are employed. Since we only have two groups in the dataset, we use *binary-metrics* to calculate *precision, recall and f1-score* over the classification results, and we also use *weighted-metrics* to account for class imbalance by computing the average of metrics in which each class score is weighted by its presence in the true data sample. Standard *precision, recall and f1-score* are considered in both scenarios. Note that weighted averaging may produce an f1 that is not between precision and recall.

#### 6.1.4 Result of United States Election 2020

The comparison results are shown in Table 2. It is not surprising that all baselines beat Random. Overall, matrix factorization methods work well for this problem. Among other baselines, Sentiment140 work poorly for this task, because 1) they use background language models that are pre-trained on another corpus; and 2) they do not user dependency information, which matters in real-world data. H-NCut and DBSCAN yield acceptable performance, but cannot compare with our BSMF algorithm with S-module, since they ignore the user dependencies. Considering weighted scores, NMTF outperforms the NMF-based algorithm, which is as expected. With the *S-module*, our BSMF algorithm ranks the top in terms of all metrics. Comparing to other variants, *M-module* in this dataset does not add benefit, mostly because several important keywords such as “president”, “Trump”, and “election” are shared by both sides. Therefore, variants using content similarity may experience confusion and not perform well. This is especially true of variants that use lexical similarity although variants that use *BERTweet* embeddings also suffer compared to BSMF_*S*_.

**TABLE 2:** Binary- and weighted- metrics comparison (United States election 2020).

Models	United States election 2020					
	Binary			Weighted		
	Prec.	Recall	F1	Prec.	Recall	F1
Random	0.655 ± 0.036	0.502 ± 0.058	0.567 ± 0.048	0.542 ± 0.036	0.487 ± 0.043	0.502 ± 0.041
Sentiment140	0.741 ± 0.000	0.208 ± 0.000	0.325 ± 0.000	0.651 ± 0.000	0.595 ± 0.000	0.530 ± 0.000
H-NCut	0.680 ± 0.002	0.980 ± 0.001	0.806 ± 0.004	0.676 ± 0.078	0.679 ± 0.006	0.566 ± 0.003
DBSCAN	0.714 ± 0.000	0.981 ± 0.000	0.827 ± 0.000	0.753 ± 0.000	0.722 ± 0.000	0.656 ± 0.000
Polarization	0.673 ± 0.002	0.982 ± 0.008	0.801 ± 0.004	0.454 ± 0.001	0.669 ± 0.006	0.541 ± 0.003
NMTF	0.679 ± 0.013	0.739 ± 0.085	0.705 ± 0.037	0.566 ± 0.018	0.588 ± 0.029	0.571 ± 0.017
FNMTF	0.747 ± 0.073	0.978 ± 0.008	0.845 ± 0.046	0.693 ± 0.167	0.753 ± 0.085	0.688 ± 0.138
BSMF	0.788 ± 0.007	0.600 ± 0.027	0.680 ± 0.020	0.677 ± 0.010	0.621 ± 0.018	0.632 ± 0.017
BSMF_*S*_	0.873 ± 0.008	0.940 ± 0.022	0.905 ± 0.013	0.867 ± 0.019	0.867 ± 0.017	0.864 ± 0.017
BSMF_*M*_	0.754 ± 0.015	0.681 ± 0.014	0.715 ± 0.010	0.655 ± 0.016	0.635 ± 0.012	0.642 ± 0.018
BSMF_*M*−*BERT*_	0.754 ± 0.015	0.724 ± 0.010	0.739 ± 0.008	0.661 ± 0.017	0.654 ± 0.013	0.657 ± 0.015
BSMF_*MS*_	0.803 ± 0.006	0.946 ± 0.035	0.869 ± 0.012	0.615 ± 0.024	0.807 ± 0.012	0.793 ± 0.007
BSMF_*MS*−*BERT*_	0.848 ± 0.007	0.964 ± 0.015	0.902 ± 0.008	0.864 ± 0.015	0.859 ± 0.011	0.852 ± 0.011

*Prec. stands for precision.

For illustrative purposes (to give a feel for the data), Table 3 shows the top 3 tweets from each belief set ( $B_{1}$ and $B_{2}$ ) estimated by our model. Note that, due to an update of the Twitter API, the crawled text field is truncated to 140 characters. Our algorithm runs on the text within that range only. For human readability and interpretability, we manually fill in the rest of the tweet, showing the additional text in yellow (the same for Table 5 and Table 7). Note that, the labels shown in the first column, called *Beliefs*, are inserted manually after the fact (and not by our algorithm). The algorithm merely does the separation/clustering.

**TABLE 3:** Top 3 tweets from separated beliefs (United States election 2020).

(Incremental) beliefs	Sample tweets
$B_{a}$ : Pro-Trump	“@ senatemajldr and Republican Senators have to get tougher, or you won’t have a Republican Party anymore. We won the Presidential Election, by a lot. FIGHT FOR IT. Don’t let them take it away!” (Dec. 18)
	The lie of the year is that Joe Biden won! Christina Bobb @OANN
	@RudyGiuliani They’ve been caught red handed! Lock them all up and declare @realDonaldTrump the winner of the presidential election!
$B_{b}:$ Anti-Trump	Trump has berserk late night meltdown and everyone just laughs at him https://t.co/Qh7uUIknux
	In other words: Donald Trump’s incompetence was the leading cause of death in the Unitd States this week. https://t.co/Gh9C0wkPIb
	Wisconsin Supreme Court turns away Trump election lawsuit https://t.co/8QvcqPgZbN https://t.co/ti9c4qJUWM

### 6.2 Star-like Belief Structure: *Eurovision2016*

Next, we consider a data set where we break sources into two subgroups but assume that they have overlapping beliefs. The example facilitates comparison with the prior state of the art on polarization that addresses a flat belief structure.

#### 6.2.1 Dataset

We use the Eurovision2016 dataset, borrowed from (Al Amin et al., 2017). Eurovision2016 contains tweets about the Ukrainian singer, Jamala, who won the Eurovision (annual) song contest in 2016. Her success was a surprise to many as the expected winner had been from Russia according to pre-competition polls. The song was on a controversial political topic, telling a story about deportation of Crimean Tatars by Soviet Union forces in the 1940s. Tweets related to Jamala were collected within 5 days of the contest. Basic statistics are reported in Table 1. As pre-processed in (Al Amin et al., 2017), the most popular 1,000 claims were manually annotated. They were separated into 600 pro-Jamala, 239 anti-Jamala, and 161 neutral claims.

In the context of the dataset, the entire set of sources is regarded as a big group, $G_{1}$ , with belief set, $B_{1}$ , agreed among all users. The group is further divided into three disjoint groups, group $G_{1a}$ (with inherited belief set $B_{1}$ and incremental belief set $B_{1a}$ ), group $G_{1b}$ (with inherited belief set $B_{1}$ and incremental belief set $B_{1b}$ ), and the residual group $G_{1}^{-}$ (with belief set $B_{1}$ ). In this case, the belief structure is:

$$B=\left[\begin{array}{ccc} 1 & 0 & 0\\ 1 & 1 & 0\\ 1 & 0 & 1 \end{array}\right]$$

where rows correspond to groups $G_{1}^{-}$ , $G_{1a}$ , and $G_{1b}$ , respectively, whereas columns correspond to belief sets, $B_{1}$ , $B_{1a}$ and $B_{1b}$ respectively.

#### 6.2.2 Baselines

We use the same baselines as in Section 6.1.2. Similarly, we also use all variants of BSMF algorithms.

#### 6.2.3 Evaluation Metrics

In this dataset, we evaluate claim separation using metrics described in Section 6.1.3. Instead of *binary-evaluation*, we use *macro-evaluation* because there are three groups in this dataset, as opposed to only two (in the previous subsection). This metric calculates the mean of the metrics, giving equal weight to each class. It is used to highlight model performance of infrequent classes. Still, note that weighted averaging may produce an *f1-score* that is not between precision and recall.

#### 6.2.4 Result of Eurovision2016

The comparison results are shown in Table 4. Similar to the results of *United States Election 2020*, all baselines beat Random, and matrix factorization methods work okay for this problem, but not as good. Sentiment140 still works poorly for the same reason as before. H-NCut and DBSCAN yield much weaker performance than before, likely because they fail to adequately consider the underlying overlapping belief structure. NMTF outperforms the NMF-based algorithm. The reason may be that its additional freedom allows it to capture the underlying structure better. With both the *M-module* and *S-module*, our BSMF algorithm ranks the top in all metrics. Both modules help in this experiment. We believe that it is due to the specific star-like belief structure and the existence of a residual group who beliefs can be better inferred by message interpolation. Further, we see that *BERTweet* variants perform better than lexical *M-modules* on several metrics in this case, because *BERTweet* is more language focused. As before, for illustration and to give a sense of the data, Table 5 shows the top 3 tweets from each belief set ( $B_{1}$ , $B_{1a}$ , $B_{1b}$ ) estimated by our model.

**TABLE 4:** Macro- and weighted- metrics comparison (eurovision 2016).

Models	Eurovision 2016					
	Macro			Weighted		
	Prec	Recall	F1	Prec	Recall	F1
Random	0.329 ± 0.016	0.326 ± 0.020	0.308 ± 0.017	0.412 ± 0.016	0.329 ± 0.017	0.350 ± 0.017
Sentiment140	0.425 ± 0.000	0.377 ± 0.000	0.339 ± 0.000	0.413 ± 0.000	0.384 ± 0.000	0.354 ± 0.000
H-NCut	0.479 ± 0.042	0.336 ± 0.001	0.245 ± 0.001	0.557 ± 0.035	0.561 ± 0.000	0.407 ± 0.001
DBSCAN	0.328 ± 0.007	0.347 ± 0.021	0.323 ± 0.008	0.462 ± 0.003	0.475 ± 0.022	0.459 ± 0.013
Polarization	0.468 ± 0.028	0.488 ± 0.025	0.438 ± 0.039	0.581 ± 0.021	0.529 ± 0.030	0.515 ± 0.032
NMTF	0.474 ± 0.056	0.450 ± 0.051	0.428 ± 0.047	0.549 ± 0.040	0.551 ± 0.053	0.522 ± 0.041
FNMTF	0.394 ± 0.012	0.348 ± 0.016	0.366 ± 0.013	0.712 ± 0.014	0.581 ± 0.033	0.636 ± 0.023
BSMF	0.521 ± 0.013	0.522 ± 0.010	0.481 ± 0.020	0.609 ± 0.016	0.523 ± 0.020	0.525 ± 0.015
BSMF_*S*_	0.628 ± 0.011	0.561 ± 0.008	0.564 ± 0.023	0.675 ± 0.006	0.675 ± 0.018	0.656 ± 0.004
BSMF_*M*_	0.584 ± 0.007	0.564 ± 0.007	0.568 ± 0.010	0.659 ± 0.006	0.628 ± 0.014	0.639 ± 0.007
BSMF_*M*−*BERT*_	0.592 ± 0.006	0.570 ± 0.009	0.576 ± 0.013	0.657 ± 0.007	0.640 ± 0.012	0.645 ± 0.004
BSMF_*MS*_	0.635 ± 0.015	0.610 ± 0.008	0.618 ± 0.010	0.697 ± 0.010	0.690 ± 0.019	0.690 ± 0.012
BSMF_*MS*−*BERT*_	0.657 ± 0.006	0.622 ± 0.009	0.635 ± 0.008	0.712 ± 0.004	0.712 ± 0.018	0.708 ± 0.009

*Prec. stands for precision.

**TABLE 5:** Top 3 tweets from separated beliefs (eurovision 2016).

(Incremental) beliefs	Sample tweets
$B_{1}$ : Agreement	BBC News–Eurovision Song Contest: Ukraine’s Jamala wins competition https://t.co/kL8SYOPOYL
	Parents of “#Ukrainian” Susana #Jamaludinova - @Jamala are #Russian citizens and prosper in the Russian #Crimea
	A politically charged ballad by the Ukrainian singer Jamala won the @Eurovision Song Contest http://nyti.ms/1qlmmNs
$B_{1a}$ : Pro-Jamala	@jamala congratulations! FORZA UKRAINE!
	@DKAMBinUkraine: Congratulations @jamala and #Ukraine!!! You deserved all the 12 points from #Denmark and the victory, #workingforDK
	@NickyByrne: Well done to Ukraine and @jamala
$B_{1b}:$ Anti-Jamala	jamala The song was political and agaisnt The song contest rules shows NATO had influence on jury decision
	@VictoriaLIVE @BBCNews @jamala Before voting we rated it worst song in the contest. Not changed my mind
	@JohnDelacour So @jamala has violated TWO ESC rules - the song is not new, and it includes political content. Result MUST be annulled

### 6.3 Joint Polarity and Topic Separation

Before we test our algorithm on a dataset with a hierarchical belief structure, we test it on something it is not strictly designed to do: namely, joint separation of topic and polarity. This problem may arise in instances where multiple interest groups simultaneously discuss different topics, expressing different opinions on them. We check whether our algorithm can simultaneously separate the different interest groups and their stances on their topics of interest. To do so, we artificially concatenated tweets from Eurovision2016 and the United States Election 2020 datasets. This operation creates a virtual hierarchy where each original topic is viewed as a different interest group. Inside each group, there are sub-structures as introduced before. Accordingly, we apply our algorithm on the following belief structure:

$$B=\left[\begin{array}{ccccc} 1 & 0 & 0 & 0 & 0\\ 1 & 1 & 0 & 0 & 0\\ 1 & 0 & 1 & 0 & 0\\ 0 & 0 & 0 & 1 & 0\\ 0 & 0 & 0 & 0 & 1 \end{array}\right]$$

where rows correspond to groups $G_{1}^{-}$ , $G_{1a}$ , $G_{1b}$ , $G_{\alpha }$ , and $G_{\beta }$ respectively, whereas columns correspond to belief sets, $B_{1}$ , $B_{1a}\;B_{1b}$ , $B_{\alpha }$ and $B_{\beta }$ respectively.

#### 6.3.1 Evaluation Metrics

We first evaluate the *accuracy* of the dataset separation by collapsing the label to each 0 and 1 corresponding to two datasets. Then, for each dataset, we employ *Macro-f1 score* and *Weighted-f1 score* on Eurovision2016 dataset, *Binary-f1 score* and *Weighted-f1 score* on United States Election 2020 dataset.

#### 6.3.2 Result of Separation

The comparison results are shown in Table 6. All BSMF variants, specifically those with *M-module* and *S-module*, performed well on separation of two datasets. In addition, comparing the *f1-scores* to the performance of the same variant in to Table 2 and Table 4, we are pleased to discover the *f1-scores* have not deteriorated, demonstrating the basic ability of our model to perform hierarchical belief estimation.

**TABLE 6:** Separation accuracy and macro-/weighted- F1 scores comparison.

Models	Separation	Eurovision 2016 F1		US election 2020 F1	
	Accuracy	Macro	Weighted	Binary	Weighted
BSMF	0.744 ± 0.017	0.451 ± 0.033	0.532 ± 0.026	0.628 ± 0.016	0.563 ± 0.010
BSMF_*S*_	0.937 ± 0.002	0.552 ± 0.038	0.625 ± 0.030	0.884 ± 0.013	0.833 ± 0.015
BSMF_*M*_	0.836 ± 0.010	0.481 ± 0.040	0.567 ± 0.052	0.681 ± 0.010	0.608 ± 0.006
BSMF_*M*−*BERT*_	0.918 ± 0.006	0.542 ± 0.022	0.605 ± 0.016	0.793 ± 0.015	0.740 ± 0.019
BSMF_*MS*_	0.952 ± 0.009	0.546 ± 0.014	0.616 ± 0.008	0.820 ± 0.025	0.767 ± 0.026
BSMF_*MS*−*BERT*_	0.952 ± 0.009	0.588 ± 0.027	0.659 ± 0.028	0.838 ± 0.017	0.780 ± 0.017

### 6.4 Hierarchical Belief Structure: *Global Warming*

We consider a real hierarchical scenario in this section, with a majority group, $G_{1}$ , and a minority group, $G_{2}$ , of beliefs $B_{1}$ and $B_{2}$ that do not overlap. Since more data is expected on $G_{1}$ (by definition of majority), we opt to further divide it into subgroups $G_{1a}$ and $G_{1b}$ , who (besides believing in $B_{1}$ ) hold the incremental belief sets $B_{1a}$ and $B_{1b}$ , respectively. The corresponding belief structure is reflected by the belief matrix:

$$B=\left[\begin{array}{cccc} 1 & 0 & 0 & 0\\ 1 & 1 & 0 & 0\\ 1 & 0 & 1 & 0\\ 0 & 0 & 0 & 1 \end{array}\right]$$

where rows represent groups $G_{1}^{-}$ , $G_{1a}$ , $G_{1b}$ , and $G_{2}$ and columns represent the belief sets $B_{1}$ , $B_{1a}$ , $B_{1b}$ , and $B_{2}$ . The matrix is not an identity matrix because beliefs overlap (e.g, groups $G_{1}^{-}$ , $G_{1a}$ , and $G_{1b}$ share belief $B_{1}$ ). It also features a hierarchical subdivision of $G_{1}$ , into $G_{1}^{-}$ , $G_{1a}$ , and $G_{1b}$ .

#### 6.4.1 Datasets

We apply this belief structure to an unlabeled dataset, Global Warming, which is crawled in real time with the *Apollo Social Sensing Toolkit*. This dataset is about a twitter discussion of global warming in the wake of Australia wildfires that ravaged the continent, in September 2019, where at least 17.9 million acres of forest have burned in the fire. Our goal is to identify and separate posts according to the above abstract belief structure.

Table 7 shows the algorithm’s assignment of claims to belief groups (only the top 3 claims are shown for space limitations). The first column shows the abstract belief categories $B_{1}$ , $B_{1a}$ , $B_{1b}$ , and $B_{2}$ . While the algorithm allocates posts to categories based on the structure of matrix **B**, for readability, we manually inspect posts assigned to each category in the matrix, and give that category a human-readable name, also shown in the first column. For each belief category, the table also shows the top ranked statements. The table reveals that sources in our data set are polarized between a group, $G_{1}$ , that believes in global warming (offering statements that urge a serious response) and a group, $G_{2}$ , that does not (offering statements that oppose the thesis of man-made global warming). Within group, $G_{1}$ (apart from $G_{1}^{-}$ ), there are two subgroups, $G_{1a}$ and $G_{1b}$ . The former blames the fossil fuel industry, whereas the latter is concerned with rising sea levels. While we do not claim to have reached conclusions on global warming, the table shows how structured matrix factorization can fit data sets automatically to useful belief structures, thereby offering visibility into what individuals are concerned with, what actions they agree on, and what they disagree about.

**TABLE 7:** Top 3 tweets from separated beliefs (global warming).

(Incremental) beliefs	Sample tweets
	Australia’s top scientists urge government to do more on global warming https://t.co/NclFqGKXE1
$B_{1}$ **:** Global Warming/urge response	Australia’s most prestigious scientific organisation has added to growing pressure on Prime Minister Scott Morrison over climate change policy, calling on the government to “take stronger action” in response to the bushfire crisis
	“Have we now reached the point where at last our response to global warming will be driven by engineering and economics rather than ideology and idiocy?” #auspol
	As long as the ALP keep accepting ‘donations’ (bribes) from the climate change deniers the fossil fuel industry, who spent millions and millions spreading lies about global warming, they have zero creditibility when they talk about phasing out fossil fuel
$B_{1a}$ **:** Global Warming/fossil fuel	To mitigate the effects of climate change, we must do away with fossil fuel burning as they are the major contributors of global warming
	Turnbull: “The world must, and I believe will, stop burning coal if we are to avoid the worst consequences of global warming. And the sooner the better.” Malcom Turnbull, The Guardian 12 January #ScottyfromMarketing
	That time when The Australian misrepresented @JohnChurchOcean to say sea level rise wasn’t linked to global warming. After I wrote about it, they pulled the story
$B_{1b}$ **:** Global Warming/sea level	Brave global warming researchers are studying sea level rise in the Maldives this morning. https://t.co/aqGtgXAj2t
	CO2 is a magical gas which causes Lake Michigan water levels to both rise and fall https://t.co/8FrC1Cx2Rm
	CLIMATE’S FATAL FLAW: ‘Greenhouse Gases Simply Do Not Absorb Enough Heat To Cause Global Warming’—“New data and improved understanding now show that there is a fatal flaw in greenhouse-warming theory.”
$B_{2}$ **:** No Global Warming	“Three new research studies confirm that geothermal heat flow, not man-made global warming, is the dominant cause of West Antarctic Ice Sheet (WAIS) melting,” writes geologist James Edward Kamis
	Left Media talks about\enleadertwodots Climate Change, Global Warming, But … Jihad is reason for recent Forest Fires in Australia !

#### 6.4.2 Quantitative Measurements

Next, we do a sanity check by measuring user grouping consistency. Specifically, we first identify the belief sets (by claim separation) and then assign belief labels to users by having a user inherit the assigned belief set label for each claim they made. The inherited labels are inconsistent if they belong to *different* groups according to matrix, **B**. For example, if the same user has been assigned belief labels $B_{1}$ and $B_{1a}$ , then the labeling is coherent because both represent beliefs of $G_{1}$ (remember that a group inherits the beliefs of its parent). If another user is labeled with both $B_{1}$ and $B_{2}$ , then it is apparently wrong, since belief sets $B_{1}$ and $B_{2}$ belong to different groups.

The percentage of coherently labeled users was 96.08%. Note that, we do not conduct comparison in this dataset, since most baselines do not uncover hierarchical group/belief structures, whereas those that do generally break up the hierarchy differently (e.g., by hierarchical topic, not hierarchical stance) thus not offering an apples to apples comparison. In future work, we shall explore more comparison options.

## 7 Related Work

The problem of modeling social groups has been long researched. For example, the problem of belief mining has been a subject of study for decades (Liu, 2012). Solutions include such diverse approaches as detecting social polarization (Conover et al., 2011; Al Amin et al., 2017), opinion extraction (Srivatsa et al., 2012; Irsoy and Cardie, 2014; Liu et al., 2015), stance detection (Darwish et al., 2020) and sentiment analysis (Hu et al., 2013a,b), to name a few.

Pioneers, like Leman *at el* (Akoglu, 2014). and Bishan *at el.* (Yang and Cardie, 2012), had used Bayesian models and other basic classifiers to separate social beliefs. On the linguistic side, many efforts to extract user opinions based on domain-specific phrase chunks (Wu et al., 2018), and temporal expressions (Schulz et al., 2015). With the help of pre-trained embedding, like Glove (Liu et al., 2015) or word2vec (Wang et al., 2017), deep neural networks (e.g., variants of RNN (Irsoy and Cardie, 2014; Liu et al., 2015)) emerged as powerful tools (usually with attention modules (Wang et al., 2017)) for understanding the polarity or sentiment of user messages. In contrast to the above supervised language-specific solutions, we want to provide options and consider the challenge of developing an *unsupervised* approach that could also be language-agnostic.

In the domain of unsupervised algorithms, our problem is different from the related problems of unsupervised topic detection (Ibrahim et al., 2018; Litou and Kalogeraki, 2017), sentiment analysis (Hu et al., 2013a,b), truth discovery (Shao et al., 2018, 2020), and unsupervised community detection (Fortunato and Hric, 2016). Topic modeling assigns posts to polarities or topic mixtures (Han et al., 2007), independently of actions of users on this content. Hence, they often miss content nuances or context that helps better interpret the stance of the source. Community detection (Yang and Leskovec, 2013), on the other hand, groups nodes by their general interactions, maximizing intra-class links while minimizing inter-class links (Yang and Leskovec, 2013; Fortunato and Hric, 2016), or partitioning (hyper)graphs (Zhou et al., 2007). While different communities may adopt different beliefs, this formulation fails to distinguish regions of belief overlap from regions of disagreement.

The above suggests that belief mining must consider both sources (and forwarding patterns) and content. Prior solutions used a source-claim bipartite graph, and determined disjoint polarities by iterative factorization (Akoglu, 2014; Al Amin et al., 2017). Our work extends a conference publication that first introduced the hierarchical belief separation problem (Yang et al., 2020). This direction is novel in postulating a more generic and realistic view: social beliefs could overlap and can be hierarchically structured. In this context, we developed a new matrix factorization scheme that considers 1) the source-claim graph (Al Amin et al., 2017); 2) message word similarity (Weninger et al., 2012) and 3) user social dependency (Zhang et al., 2013) in a new class of non-negative matrix factorization techniques to solve the hierarchical overlapping belief estimation problem.

The work also contributes to non-negative matrix factorization. NMF was first introduced by Paatero and Tapper (Paatero and Tapper, 1994) as the concept of positive matrix factorization and was popularized by the work of Lee and Seung (Lee and Seung, 2001), who gave an interesting interpretation based on parts-based representation. Since then, NMF has been widely used in various applications, such as pattern recognition (Cichocki et al., 2009) and signal processing (Buciu, 2008).

Two main issues of NMF have been intensively discussed during the development of its theoretical properties: solution uniqueness (Donoho and Stodden, 2004; Klingenberg et al., 2009) and decomposition sparsity (Moussaoui et al., 2005; Laurberg et al., 2008). By only considering the standard formula ***X ˜ UM*** ^*⊤*^, it is usually not difficult to find a non-negative and non-singular matrix **V**, such that **UV** and **V** ^**−1**^ **M** ^***⊤***^ could also be a valid solution. Uniqueness will be achieved if **U** and **M** are sufficiently sparse or if additional constraints are included (Wang and Zhang, 2012). Special constraints have been proposed in (Hoyer, 2004; Mohammadiha and Leijon, 2009) to improve the sparseness of the final representation.

Non-negative matrix tri-factorization (NMTF) is an extension of conventional NMF (i.e., ***X ˜ UBM*** ^***⊤***^ (Yoo and Choi, 2010)). Unconstrained NMTF is theoretically identical to unconstrained NMF. However, when constrained, NMTF possesses more degrees of freedom (Wang and Zhang, 2012). NMF on a manifold emerges when the data lies in a nonlinear low-dimensional submanifold (Cai et al., 2008). Manifold Regularized Discriminative NMF (Ana et al., 2011; Guan et al., 2011) were proposed with special constraints to preserve local invariance, so as to reflect the multilateral characteristics.

In this work, instead of including constraints to impose structural properties, we adopt a novel belief structured matrix factorization by introducing the mixture matrix **B**. The structure of **B** can well reflect the latent belief structure and thus narrows the search space to a good enough region.

## 8 Conclusion

In this paper, we discuss computational modeling of polarized social groups using a class of NMF, where the structure of parts is already known (or assumed to follow some generic form). Specifically, we use a belief structure matrix **B** to describe the structure of the latent space and evaluate a novel *Belief Structured Matrix Factorization* algorithm (BSMF) that separates overlapping, hierarchically structured beliefs from large volumes of user-generated messages. The factorization could be briefly formulated as **X** ^*MS*^ ≈ **UBM** ^*⊤*^, where **B** is known. The soundness of the model is first tested on a synthetic dataset. A further evaluation is conducted on real Twitter events. The results show that our algorithm consistently outperforms baselines. The paper contributes to a research direction on automatically separating data sets according to arbitrary belief structures to enable more in-depth modeling and understanding of social groups, attitudes, and narratives on social media.

## Data Availability

The original contributions presented in the study are included in the article/Supplementary Material, further inquiries can be directed to the corresponding author.

## Footnotes

1 In practice, we permute the labels and pick the best matching as a result, since our approach does clustering not classification.

2
http://apollo4.cs.illinois.edu/datasets.html
